# QTL analysis on a lemon population provides novel insights on the genetic regulation of the tolerance to the two-spotted spider mite attack

**DOI:** 10.1186/s12870-024-05211-4

**Published:** 2024-06-07

**Authors:** Chiara Catalano, Mario Di Guardo, Giuliana Licciardello, Sebastiano Seminara, Giovanna Tropea Garzia, Antonio Biondi, Michela Troggio, Luca Bianco, Stefano La Malfa, Alessandra Gentile, Gaetano Distefano

**Affiliations:** 1 Department of Agriculture, Food and Environment, University of Catania, Via Santa Sofia 100, Catania, 95123 Italy; 2 Research and Innovation Centre, San Michele All’ Adige, Fondazione Edmund Mach, Trento, Italy

**Keywords:** *Citrus limon*, *Tetranychus urticae*, Marker-assisted selection (MAS), Single-primer enrichment technology, Ethylene-responsive transcription factor ERF098-like

## Abstract

**Background:**

Among the *Citrus* species, lemon (*Citrus limon* Burm *f.*) is one of the most affected by the two-spotted spider mite (*Tetranychus urticae* Koch). Moreover, chemical control is hampered by the mite’s ability to develop genetic resistance against acaricides. In this context, the identification of the genetic basis of the host resistance could represent a sustainable strategy for spider mite control. In the present study, a marker-trait association analysis was performed on a lemon population employing an association mapping approach. An inter-specific full-sib population composed of 109 accessions was phenotyped through a detached-leaf assays performed in modified Huffaker cells. Those individuals, complemented with two inter-specific segregating populations, were genotyped using a target-sequencing approach called SPET (Single Primer Enrichment Technology), the resulting SNPs were employed for the generation of an integrated genetic map.

**Results:**

The percentage of damaged area in the full-sib population showed a quantitative distribution with values ranging from 0.36 to 9.67%. A total of 47,298 SNPs were selected for an association mapping study and a significant marker linked with resistance to spider mite was detected on linkage group 5. I*n silico* gene annotation of the QTL interval enabled the detection of 13 genes involved in immune response to biotic and abiotic stress. Gene expression analysis showed an over expression of the gene encoding for the ethylene-responsive transcription factor ERF098-like, already characterized in *Arabidopsis* and in rice for its involvement in defense response.

**Conclusion:**

The identification of a molecular marker linked to the resistance to spider mite attack can pave the way for the development of marker-assisted breeding plan for the development of novel selection coupling favorable agronomical traits (e.g. fruit quality, yield) with a higher resistance toward the mite.

**Supplementary Information:**

The online version contains supplementary material available at 10.1186/s12870-024-05211-4.

## Introduction

Within the *Tetranychidae* family, *Tetranychus urticae* Koch and *Panonychus citri* Mc Gregor pose significant threats to citriculture in the Mediterranean basin [1], particularly affecting clementines and lemons. Feeding punctures on the leaves lead to leaf spot chlorosis, resulting in photosynthesis impairment. Sever infestations may also cause phylloptosis and scarring on the fruit. To preserve crop performance and fruit marketability, spider mite control integrates specific agronomical practices (e.g., the use of ground cover), biological control, and the rational use of pesticides by alternating the active principles according to the Insecticide Resistance Action Committee (IRAC) indications [2]. The reliance on chemical pesticides alone is no longer an effective and sustainable solution for spider mite control. The spread of this pest was also favored by the simultaneous occurrence of the *Citrus Tristeza Virus* (CTV). This was exacerbated by the replacement of sour orange, a rootstock characterized by a high susceptibility to CTV, with CTV-resistant rootstocks that are often characterized by a greater susceptibility to *T. urticae* [3]. Bruessow and colleagues [3] demonstrated the influence of rootstock on fecundity, oviposition rate, lifespan, and development time. In particular, Alemow (*Citrus macrophylla*), Volkamer lemon (*Citrus volkameriana*), and ‘Cleopatra’ mandarin (*Citrus reshni*) exhibited the highest susceptibility to the spread of *T. urticae*, while, as expected, sour orange was the least suitable for the spider mite survival, followed by Troyer citrange (*Citrus sinensis* ‘Washington’ x *Poncirus trifoliata*) and trifoliate orange (*P. trifoliata*). In 2016, Agut and colleagues [4] confirmed the rootstock influence on scion resistance against biotic stresses, demonstrating the negative impact on spider mite oviposition when *Citrus clementina* was grafted onto sour orange. Transcriptomic and metabolomic analyses of the root efflux revealed a strong accumulation of glutamic acid, triggering the expression of the putative citrus glutamate receptor in the shoots. Previous studies on *C. clementina* highlighted that spider mite induces changes in the proteomic profile of the attacked plants, triggering an overexpression of defense-related proteins (chitinase, a lectin-like protein, the protease inhibitor miraculin-like protein, heat shock proteins, and lipoxygenases) [5, 6]. A successive study coupling hormonal, metabolomic, and transcriptomic approaches on sour orange and ‘Cleopatra’ mandarin (respectively resistant and susceptible to spider mite) highlighted the role of oxylipin, jasmonic acid, and flavonoids in the plant-host interaction [7]. The production of Herbivore-Induced Plant Volatiles (HIPVs) were investigated in the aforementioned genotypes, and it was demonstrated that terpenes such as α-ocimene, α-farnesene, pinene and D-limonene, and the green leaf volatile 4-hydroxy-4-methyl-2-pentanone (highly expressed in sour orange) have a marked repellent effect on conspecific mites, while volatiles such as 2-butoxyethoxy ethanol, benzaldehyde, and methyl salicylate (expressed in ‘Cleopatra’ mandarin) promote mite attraction [8]. It is worth noticing that research focused on resistance towards spider mite attack in citrus was mainly conducted by investigating metabolomic or proteomic changes in rootstock genotypes. To date, to the best of our knowledge, there are no reports on quantitative trait *loci* (QTLs) associated with the trait of interest, although more recently QTL analysis through high-throughput genotyping is one of the most applied strategies for the identification of molecular markers and candidate genes related to important traits, such as fruit quality [9], biotic [10] and abiotic [11] stress.

In the present research, a segregating population of lemon was developed to study the genetic determinism of the resistance/susceptibility to two-spotted spider mite attack, by crossing lemon (*Citrus limon*) varieties ‘Interdonato’ (♀) and ‘Femminello Siracusano 2Kr’ (♂). The phenotyping of the population involved the evaluation of the area of damaged leaves and the mite survival rate in detached-leaf bioassays. The phenotyping method was initially validated on six reference citrus genotypes together with the two parents of the segregating population. Genotyping was performed through a target-sequence approach, and phenotypic and genotypic data were integrated for an association mapping analysis leading to the identification of a quantitative trait locus (QTL) strongly associated with the area of infection and validated through a gene expression analysis.

## Materials and methods

### Constitution of the segregating lemon populations

In May 2018, cross pollination between *C. limon* ‘Interdonato’ (♀) and *C. limon* ‘Femminello Siracusano 2Kr’ (♂) lemons was performed. Both parents represent two of the most widely employed lemon cultivars in Italy and are characterized by a different response to spider mite attack. Although lemon is generally considered polyembryonic, meaning that alongside the zygotic embryo of sexual origin there could be one or more nucellar embryos originated from maternal tissue, there is a high variability in this trait among lemon cultivars [12]. The female parent of the population ‘Interdonato’ showed a very low percentage of polyembryonic seeds compared to the most widely cultivated lemon in Italy and worldwide [12], indicating that seedling obtained developed only from a zygotic embryo. The pollinated flowers were wrapped in paper bags to prevent accidental cross-pollination. In the fall of the same year, 40 ‘Interdonato’ fruits were collected, and seeds (around 200) were extracted, soaked in distilled water, and sown in peat pellets in a climate chamber (25 ± 1 °C, 40–60% RH and 16:8 h – light: dark – photoperiod). After two months, germinated seedlings were transferred to 15 cm cylindrical pots containing peat and perlite (3:1 v/v) to grow as needed for grafting propagation. Finally, the mother plants of the segregating lemon population (102 individuals) were grafted onto ‘Alemow’ rootstock (*C. macrophylla*) and held in 30 L cylindrical pots. Before assays, the plants were cultivated under an anti-insect net and maintained free from pests. Broad-spectrum insecticides were applied up to 30 days before the assays, then natural enemies (e.g. *Phytoseiulus persimilis*) were released for the biological control of the two-spotted spider mite. Two additional segregating populations (for a total of 255 accessions) and the four ancestors of lemon (*Citrus maxima, Citrus reticulata, Citrus medica and Citrus aurantium*; [13]) were also included in the study for the generation of the linkage map. The two families were inter-specific crosses both having ‘Femminello Siracusano 2Kr’ as male parent and *Citrus latipes* or *Citrus clementina* as female parents as further detailed in Di Guardo et al. (2023) [14].

### Phenotyping for spider mite resistance

**Fig. 1:**
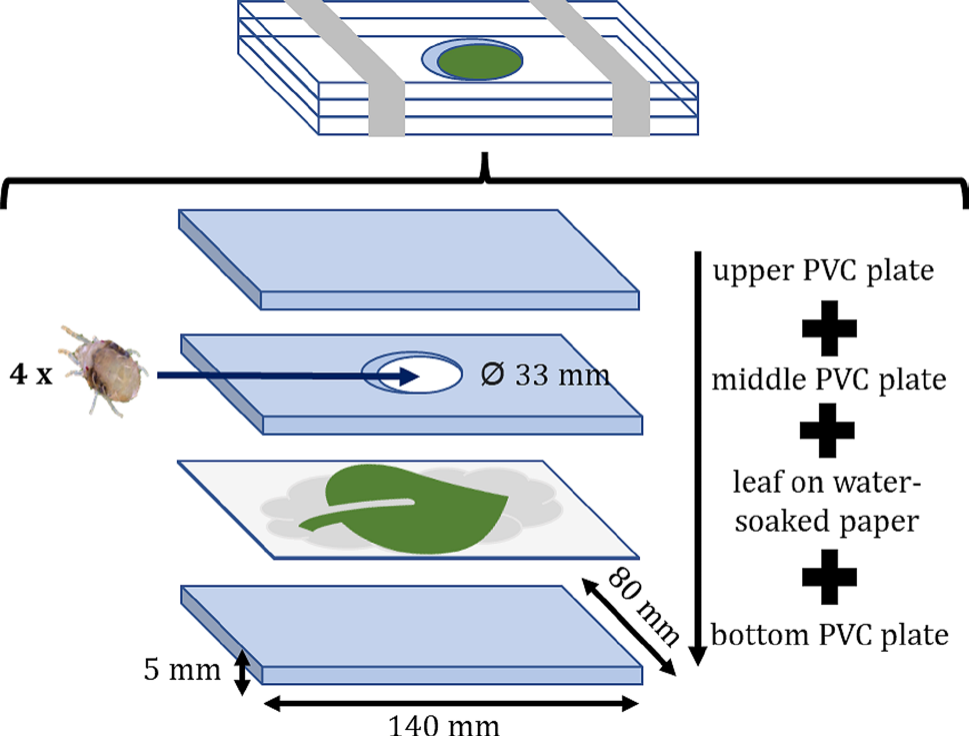
Scheme of the modified Huffaker cell used in the phenotyping assay for the attack of the two-spotted spider mite

Phenotyping assays were performed on the 102 individuals of the lemon segregating population, on the two parental lines (lemon ‘Interdonato’ and lemon ‘Femminello Siracusano 2Kr’), and reference citrus genotypes selected based on previous research on spider mite attack on citrus [3]. The selected references include sour orange (*C. aurantium*, resistant), Carrizo and Troyer citrange (*C. sinensis* ‘Washington’ x *P. trifoliata*, mildly resistant), trifoliate orange (*P. trifoliata*, mildly tolerant), Alemow (*C. macrophylla*, susceptible), and ‘Cleopatra’ mandarin (*C. reshni*, susceptible). Resistance to *T. urticae* attack was assessed through a detached-leaf system using a modified Huffaker cell [3]. The cell consisted of PVC plates arranged as follows: (1) a PVC plate (140 mm x 80 mm x 5 mm) at the bottom, (2) a damp sheet of paper, (3) the tested leaf with the bottom surface facing up, (4) another PVC plate with a central hole (Ø 33 mm), and (5) a second full PVC plate (Fig. 1). Four young leaves per plant were freshly collected and placed in the experimental arenas. A mite colony, originating from a fallow field and maintained on tomato seedlings, was used in the trials. Four one-day-old female spider mites were released on the abaxial leaves surface of each leaf, then Huffaker cell layers were closed and placed upside down in a climate chamber (25 ± 1 °C, 30–40% RH and 16:8 h – light: dark – photoperiod). Four days after the mite release, their survival was assessed, while seven days after the beginning of assays leaves were scanned (EPSON Perfection 4180 Photo scanner, 24-bit color, 2400 dpi). The timings for mite feeding and leaf damage assessment were chosen in order to ensure a standardized mite survival and feeding among the arenas (according to preliminary trials, the mite survival at the fifth day started decreasing considerably) and to have evidence of damaged area that was not visible four days after the mite release. The damaged area was selected digitally assessed with Adobe Photoshop 2021 (v. 22.2.4) and its percentage was calculated in respect of the total area of the experimental arena. Descriptive statistical analyses (mean, standard deviation, standard error), histograms and density plots were performed using the ‘stat’ and the ‘ggplot2’ packages of the R software, respectively [15, 16].

### Genotyping and QTL analysis

For genotyping, the DNA of the 361 accessions was extracted from young leaves through the QIAGEN DNeasy Plant Mini Kit following manufacturer’s instructions. DNA quantity and quality were assessed with NanoDropTM 2000c (ThermoFisher Scientific) before sequencing. Even though the work is based on the analysis of the crossing population ‘Interdonato’ x ‘Femminello Siracusano 2Kr’, two additional populations having ‘Femminello Siracusano 2Kr’ as female parent were genotyped and included in the study for the generation of the linkage map. The choice of including two additional families resulted in a much higher mapping resolution in light of the significant increase in the number of meiosis analyzed. The three segregating populations and the samples composing the pedigree of lemon were genotyped with a target sequencing approach called Single Primer Enrichment Technology (SPET) [17]. SNP detection involved resequencing a discovery panel comprising the four parental lines employed for the generation of the segregating populations. The sequencing was performed at a read depth of 80X using Illumina PE 150 technology and aligned against the lemon reference genome [Di Guardo et al. in preparation]. The linkage map was generated using LepMAP3, a software enabling the generation of integrated map through the simultaneous analysis of multiple segregating-families connected through a pedigree [18]. LepMAP3 provides a pipeline composed of five modules: ParentCall2, Filtering2, SeparateChromosomes2, JoinSingles2All, and OrderMarkers2. ParentCall2 computed genotype likelihoods from a variant calling format (vcf) file and a pedigree file; then markers with skewed segregations were filtered out with the Filtering2 module with default values (dataTolerance = 0.01). SeparateChromosomes2 performs the grouping of the markers, a LOD values ranging from 2 to 30 was computed and then additional SNPs were included in the linkage groups using the JoinSingles2All module. For the selected linkage groups, marker ordering was performed with the OrderMarkers2 module and, for each of the selected linkage groups, the ordering was computed five times to select the run with the best likelihood. SNP phasing was calculated using FlexQTL software (https://www.wur.nl/en/show/flexqtl.htm; [19]) and markers showing double recombination were manually positioned or removed (unpublished data). The occurrence of SNP within coding regions was assessed using the SNPeff software [20]. The marker-trait association analysis was carried out using the R package rrBLUP [21] and p values were corrected for multiple testing using the false discovery rate correction method (FDR) as implemented in the p.adjust function of the R software. The association mapping was based on the following mixed model:

$$\varvec{Y}= \varvec{\mu }+\varvec{X}\varvec{\beta }+\varvec{Q}\varvec{v}+\varvec{Z}\varvec{u}+\varvec{\varepsilon }$$

Where Y is the vector of phenotypic values, µ the overall mean, X the matrix of the genotypes and β the allelic effect of each SNP, Q is the matrix of the stratification within the population (genetic structure) and v the effect vector estimated by the model, Z is an incidence matrix linking observations to the vector u (kinship) and ε the residual effect. Qv is considered as fixed effect while Zu as random effect. Gene annotation was carried out using the lemon reference genome (https://www.citrusgenomedb.org/Analysis/1462349) with an in-house R script (available upon request). The list of candidate genes was further investigated through gene ontology prediction and functional annotation using the eggNOG software (http://eggnog5.embl.de/#/app/home) [22] The REVIGO software (http://revigo.irb.hr/) [23] was used to visualize gene ontology relationship through network analysis.

### Gene expression analysis

For gene expression analysis, genotypes at the extremes of the phenotypic evaluation scale for tolerance towards spider mite attack, expressed as a percentage of damaged area, were selected. Four young leaves per each genotype were collected and subjected to spider mite infestation, following the procedures outlined in Fig. 1. Seven days after the spider mite release, leaves were collected and immediately ground in liquid nitrogen, and then three aliquots of 100 mg of the homogenized material were used for RNA extraction using RNeasy extraction kit (Qiagen) according to manufacturer’s protocol. On-column DNase treatment was also performed using RNase-Free DNase Set (Qiagen) according to the manufacturer’s protocol, and RNA was eluted in 30 µl of RNase- and DNase-free water. RNA quality and quantity were assessed through gel electrophoresis (1.0% agarose in TBE 1x) and Nanodrop 2000c spectrophotometer (Thermo Scientific), respectively. The synthesis of cDNA was carried out using the High-Capacity cDNA Reverse Transcription Kit (Thermo Fisher). Specifically, 500 ng of RNA was combined with 1× RT Buffer, 4 mM of dNTPs, 1× RT Random Primers, 0.1× of MultiScribe Reverse Transcriptase in a final volume of 20 µL. Thermal cycler conditions for cDNA synthesis were as follows: 10 min at 25°C, 37°C for 120 min, and 85°C for 5 s. RT-PCR was carried out using the Rotor-Gene Q thermal cycler (Qiagen). The PCR mixture contained 50 mM MgCl_2_, 1x NH_4_, 5 µM dNTPs, 50 µM SYTO-9, 0,15 units of Taq polymerase, 10 µM of each gene-specific forward and reverse primer, and 100 ng of the cDNA sample, in a final volume of 15 µl. The standard thermal profile was used for all PCRs and consisted of 95°C for 5 minutes, followed by 40 cycles at 95°C for 5 seconds,59°C for 20 seconds, 72°C for 2 minutes, 95°C for 1 minute, 40°C for 1 minute, melting from 60°C to 92°C holding 2 seconds between each 0.2°C temperature step. The melting curve was useful for excluding the formation of nonspecific amplicons and dimers since only one pick was observed for each gene. Two technical replicates were assayed for each biological replicate, and a no-template negative control was routinely included in each reaction. Primers were designed using Primer 3 software [24] (Table 1). The elongation factor 1 alpha (Cs8g16990) served as a housekeeping gene to normalize the expression data (forward: 5’ATTGACAAGCGTGTGATTGAGC3’, reverse: 5’TCCACAAGGCAATATCAATGGTA3’). Data analysis was carried out using the normalized 2^−ΔΔCT^ method, and qRT-PCR results between genotype groups were compared according to the normalized Ct value for each gene. Results are also discussed in terms of ‘mRNA fold-increase’ with respect to the calibrator, represented by the group of genotypes in the lemon segregating population considered as susceptible according to the phenotyping assay. Statistical analyses were conducted using the ‘stat’ package of the R software [15] and involved the calculation of standard deviation and standard error for each group in each gene.

**Table 1:** Names, coordinates, functions, and associated gene ontology terms of the candidate genes detected in the QTL interval on linkage group 5. Only genes whose ontology term was related to response to biotic stress and/or synthesis of primary or secondary metabolites were displayed

Gene	Start position	End position	Length (bp)	Function	Forward primer (5’->3’)	Reverse primer (5’->3’)	Amplicon (bp)
CL5G020217011.t1_pri	37,572,680	37,575,810	3131	CDT1-like protein	TTTCTGCCAGCTCACCAAAC	CGTCGCATTCTTCACAGGAG	95
CL5G020245011.t1_pri	37,722,990	37,725,758	2769	DNA-directed RNA polymerase III subunit RPC8	GGTGGTGTTTCGTCCCTTTC	TTGGACATACAGCGCAAACC	84
CL5G020255011.t1_pri	37,825,066	37,829,444	4379	NADH dehydrogenase [ubiquinone] flavo-protein 2	TCGACAGAGAAGGGAGTTGG	GTGACCATCAGTTGCACACC	83
CL5G020257011.t1_pri	37,831,544	37,835,722	4179	transcription factor UNE12	CGCATTGCACTCTTCCTCTC	CATTGCTGGTGGATGTGGAG	118
CL5G020209011.t1_pri	37,855,313	37,863,445	8133	tryptophan synthase alpha chain-like	GCAGGAGAGAGGATGATGCT	TGGACCAATGCAAACCACTG	93
CL5G020210011.t1_pri	37,533,194	37,533,640	447	ethylene-responsive transcription factor ERF098-like	GCAGCTGAAATACGTGACCC	AAGGCAAAAGCAGCTCTGTC	107
CL5G020213011.t1_pri	37,547,942	37,548,916	975	RING-H2 finger protein ATL16	TGGATTCTGCAGCTGATCGA	AAAGATTTGCGGACTCTGGC	118
CL5G020214011.t1_pri	37,550,957	37,553,310	2354	protein KINESIN LIGHT CHAIN-RELATED 3	AAAGACAGCAATGGGGAGGA	TCGCCACTCCTAATTCCCTG	107
CL5G020216011.t1_pri	37,564,267	37,569,020	4754	autophagy-related protein 9	GCTTTTGAGCCTGGGTTTCA	CTTTGCATTTCGGAGGCCAT	82
CL5G020222011.t1_pri	37,592,040	37,598,418	6379	transcription factor bHLH155	CATGAAGCGTTAGGACCTGC	GTCAACTCAGGCATCCCAAC	101
CL5G020235011.t1_pri	37,675,071	37,677,000	1930	WRKY transcription factor 21	TGAGTCAGCCCCAAGATCAG	CACTCTTGCATGACCCAACC	119
CL5G020239011.t1_pri	37,694,126	37,698,371	4246	E3 ubiquitin protein ligase DRIP2	GGCGAGCAATAATCTGGTGG	ATATGGTGGTGGCATCCCTC	101
CL5G020250011.t1_pri	37,782,849	37,792,493	9645	F-box/LRR-repeat protein 17	CAACCCAAGAAAGGCCACTC	ATAGCAGCGGAAGAATCGGA	95

## Results

### Phenotyping of the reference genotypes

**Fig. 2:**
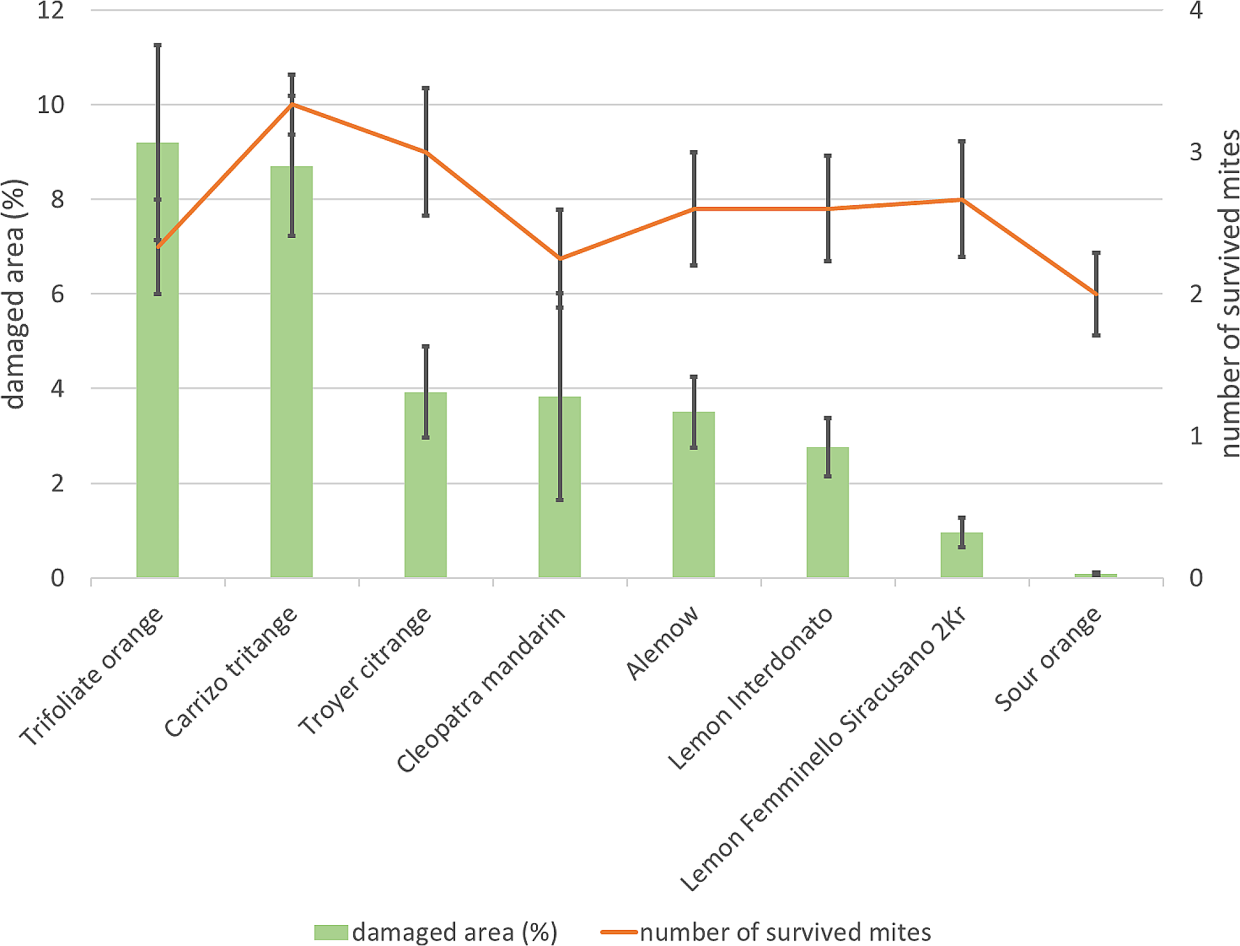
Results of phenotyping towards the attack of the two-spotted spider mite on the selected reference genotypes

Among the reference genotypes, trifoliate orange (9.2%) and Carrizo citrange (8.7%) exhibited the highest percentage of damaged area, followed by Troyer citrange (3.9%), ‘Cleopatra’ mandarin (3.8%), and Alemow (3.5%), Fig. 2. The two parental lines showed a statistically significant difference in the damaged area (t test, p value = 0.02) with values of 2.75% for ‘Interdonato’ and 0.96% for ‘Femminello Siracusano 2Kr’. Among the genotypes tested, sour orange displayed the lowest degree of damage (0.08%). Four days after the beginning of the assays, the number of survived mites was also recorded: Carrizo and Troyer citrange showed the highest values, 3.3 and 3.0 respectively, followed by ‘Femminello Siracusano 2Kr’ lemon (2.6), Alemow (2.6), ‘Interdonato’ lemon (2.6), trifoliate orange (2.3), ‘Cleopatra’ mandarin (2.2), and sour orange (2.0), Fig. 2. The percentage of damaged area and the number of survived mites during the experiment exhibited a positive moderate correlation (0.43), as a potential consequence of the low variability in mite survival among the genotypes and high variability in the damage. Overall, the citrus accessions were characterized by a wide range of response against the two-spotted spider mite attacks. The lemons ‘Interdonato’ and ‘Femminello Siracusano 2Kr’, both showing moderate tolerance to the pest, were still characterized by significant difference in the symptom development.

### Phenotyping of the lemon segregating population towards the attack of the two-spotted spider mite

**Fig. 3:**
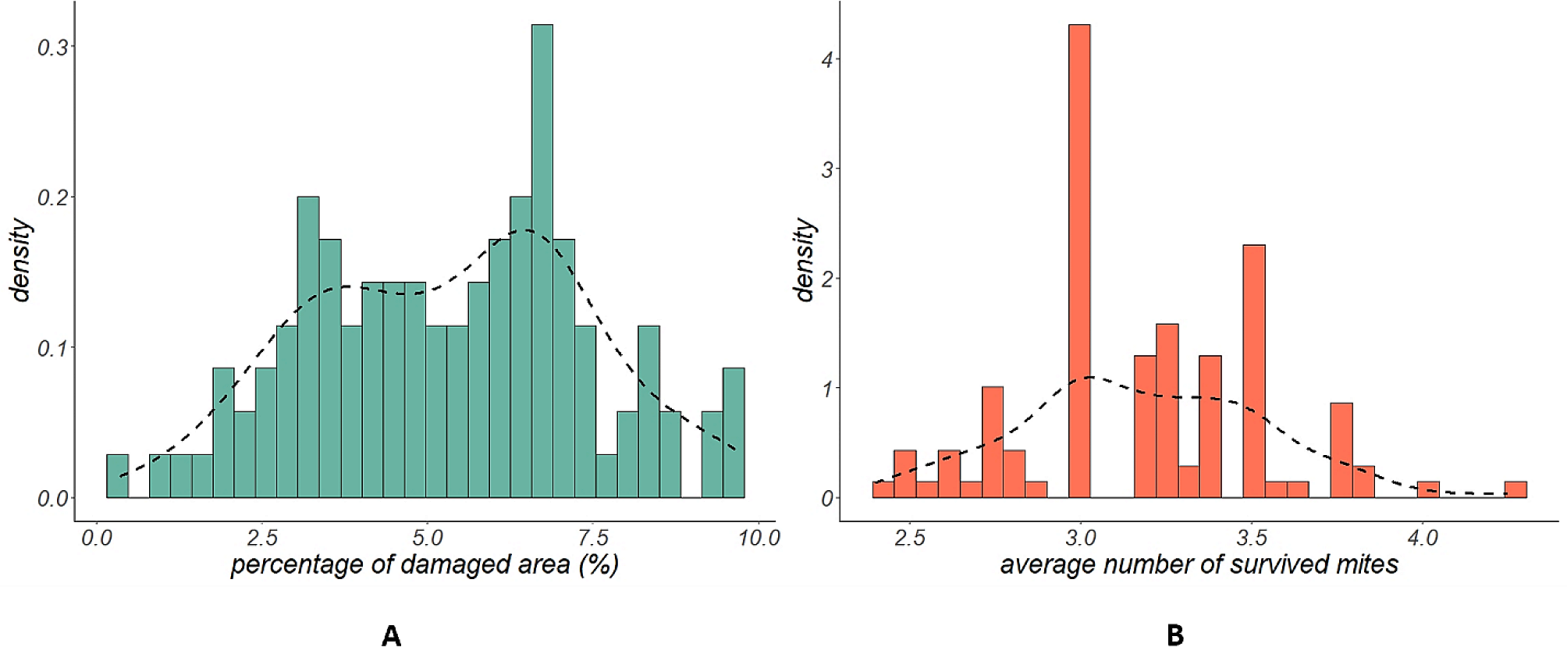
Histograms and density plot showing the phenotypic variability for the mean percentage of damaged area (**A**) and the average number of survived mites (**B**) calculated for individuals of the population ‘Interdonato’ x ‘Femminello Siracusano 2Kr’

**Fig. 4:**
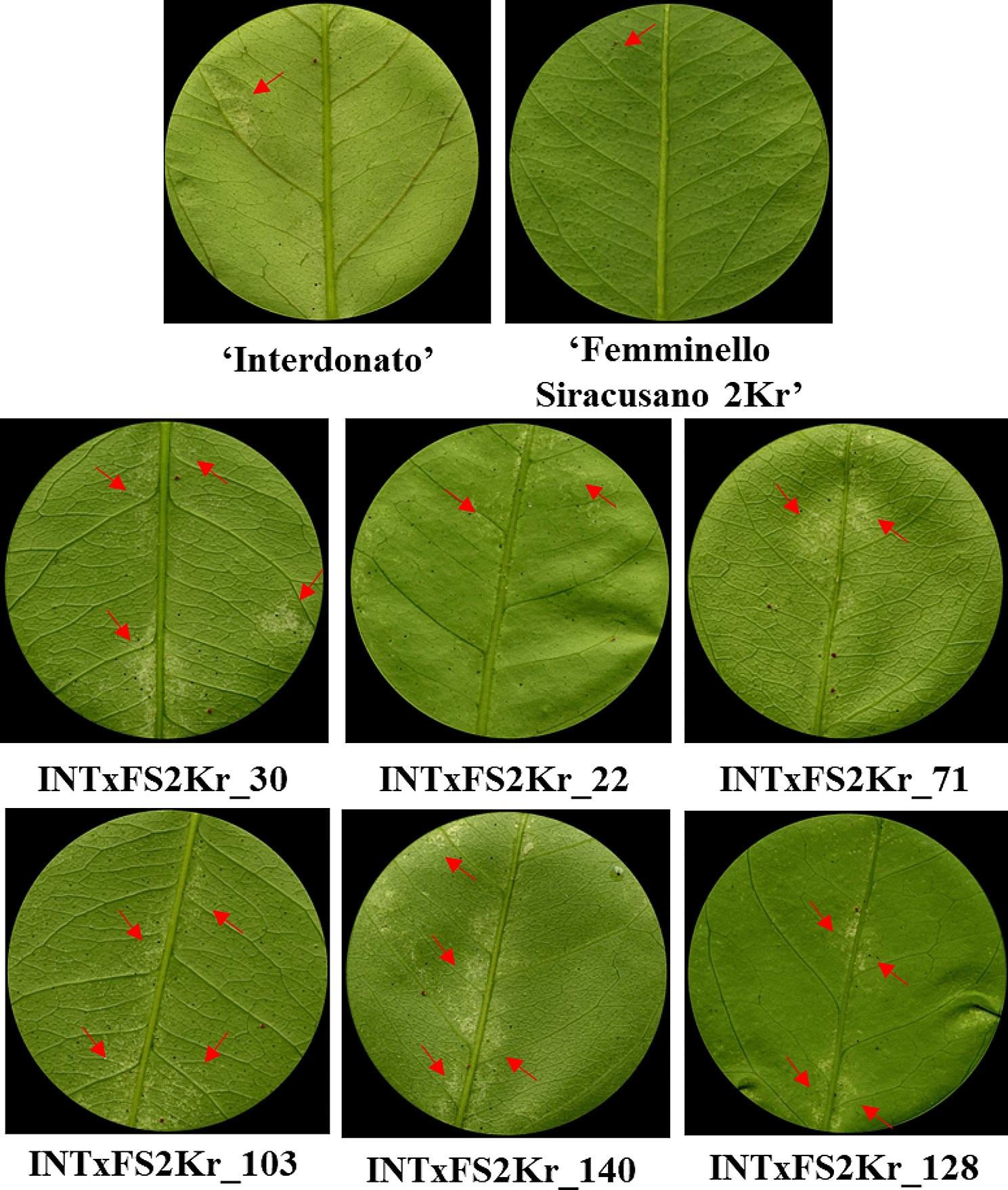
Symptoms of spider mite infestation on leaves of the parental lines of the full-sib population and in some interspecific hybrids, red arrows indicate yellow spots caused by mite feeding

Detached-leaf assays were performed to assess the behavior towards the two-spotted spider mite attack on the 102 genotypes of the lemon segregating population. At least four replicates were tested per each genotype, totaling 408 assayed leaves. The individuals of the full-sib population were characterized by a great variability in both the percentage of damaged area and number of survived mites (Fig. 3). The histogram and density plots in Fig. 3A display, for the percentage of damaged area, a minimum average value of 0.36%, almost three times lower than the parental line ‘Femminello Siracusano 2Kr’, and a maximum of 9,67%, which is greater than what obtained in trifoliate orange, the most susceptible reference genotype tested during the assays and four times higher than ‘Interdonato’, the parental line showing the highest susceptibility to the pest. The median value for the percentage of damaged area was 5.5%. Four days after the release of spider mite, Huffaker cells were checked to assess spider mite survival and globally evaluate leaf status. In the lemon segregating population, the number of survived mites ranged from a maximum of 4.2 to a minimum of 2.4, with a median value of 3.2. (Fig. 3B; Additional file 1). Is not surprising that, in a very few experimental arenas (0.96%), survived mites were more than those to be released (four) for two main reasons: firstly, we cannot exclude the accidental release of more than four mites (either adults or small young instars), secondly, it was possible that eggs early oviposited by freshly released mites in the cell could hatch and give rise to new mites (young instars). As shown in Fig. 4 and in Additional file 2, the main damage caused by spider mite is the yellowish areas representing the typical chlorosis due to mite feeding. In fact, the two-spotted spider mite has needle-like piercing-sucking mouthparts able to penetrate mesophyll cells to ingest their contents. The whitish speckles were mainly detected alongside the central leaf vein or along the experimental arena perimeter, as we observed that mites always try to find a hole or a crack as an escape route out of the Huffaker cell. Both phenotypic traits were then analyzed in association with the genotypic data for a marker-trait association analysis.

### Genotyping of the full-sib populations and generation of the genetic map

The analysis of the raw sequencing data of the four parental lines enabled the detection of more than 2 million SNPs. Then 50.000 loci were selected for the SPET genotyping based on their genetic position (to ensure an even coverage of the whole genome), and on the genotypic calls of the parental lines (enabling a uniform selection of SNPs segregating in the three segregating populations). Furthermore, SNPs located within coding regions were prioritized. The SPET genotyping on the segregating populations resulted in the detection of 424,528 SNPs, then a filtering pipeline based on the SNP quality call, the number of missing data, and the occurrence of parent-child inconsistencies allowed the detection of 55,010 SNPs that were used for the generation of an integrated linkage map (Di Guardo et al. submitted). After mapping, 7,638 SNPs were further discarded due to segregation errors, resulting in a final set of 47,298 ‘robust’ SNPs (Additional file 3). The total length of the map was 1050.3 cM, with the size of the linkage groups ranging from 70.8 cM (LG1) to 156 cM (LG5) and a number of markers for linkage group ranging from 10,968 (chr. 5) to 1,018 (chr. 4); Additional file 4. This set of SNPs was employed both to confirm the zygotic origin of all the individuals composing the segregating population and to perform the downstream marker-trait association analysis.

### Association mapping analysis and *in silico* gene annotation

**Fig. 5:**
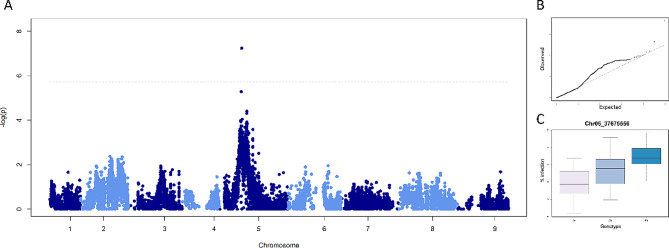
Association mapping analysis on the resistance to the attack of the two-spotted spider mite. (**A**): Manhattan plot, where the dashed horizontal line represents the significance threshold (p value = 0.05 after correction for multiple testing). (**B**): Quantile distribution of the observed p-values (y-axis) versus the quantile distribution of the expected p-values (quantile-quantile plot). (**C**): Boxplot of the phenotypic distribution of the full-sib family grouped according to the genotypes at marker Chr05_37675556 (which exhibits the highest association with the analyzed trait)

**Fig. 6:**
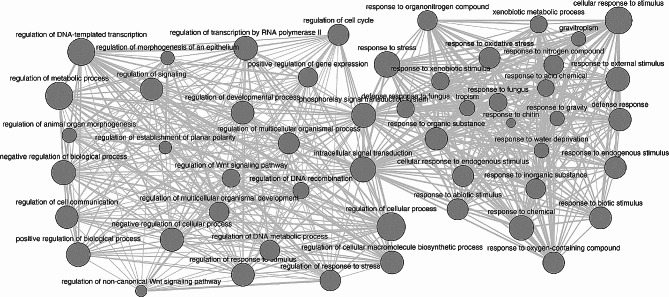
Network analysis based on the gene ontology terms of the biological processes linked to the response to spider mite attack

Phenotypic and genotypic data were integrated in an association mapping approach. While the analysis on the survived mites did not highlight any significant QTLs, the percentage of damaged area showed a significant marker-trait association on chromosome 5 (Fig. 5A-B), with the marker Chr05_37675556 (50.1 cM, -log10 of the p value = 7.2) showing the highest statistical significance (Fig. 5C). The phenotypic distribution according to the three genotypes defined by Chr05_37675556 showed a mean percentage of damaged area of 3.67 and 6.9 for AA and BB genotypes respectively with the heterozygous genetic configuration characterized by an intermediate phenotypic value (5.3, Fig. 5C). A genomic window spanning from the flanking markers, Chr05_37824975 (49.3 cM, -log10 p value = 5.2), and Chr05_37421847 (51.4 cM, -log10 p value = 4) was selected for *in silico* annotation of the QTL region to detect candidate gene(s) associated to the resistance/susceptibility to two-spotted spider mite attack. The analysis of the reference lemon genome [25] enabled the identification of 37 genes within the detected QTL region. A gene ontology analysis (Fig. 6) identified a subset of 13 genes associated with the production of metabolites and/or the response to biotic stress as further detailed in Table 1. In particular, among the annotated genes, some have been previously investigated for their involvement in plant immunity, immune signaling response, and resistance against a wide spectrum of biotic and abiotic stress, such as E3 ubiquitin-protein ligase (CL5G020239011.t1_pri) [26–28] and NADH dehydrogenase ubiquinone flavoprotein 2 (CL5G020255011.t1_pri) [29]. Overall, gene mapping and annotation enabled the selection of 37 genes within two QTL regions in chromosome 5 and a subset of 13 genes associated with metabolites production was implemented in gene expression analysis.

### Gene expression analysis

**Fig. 7:**
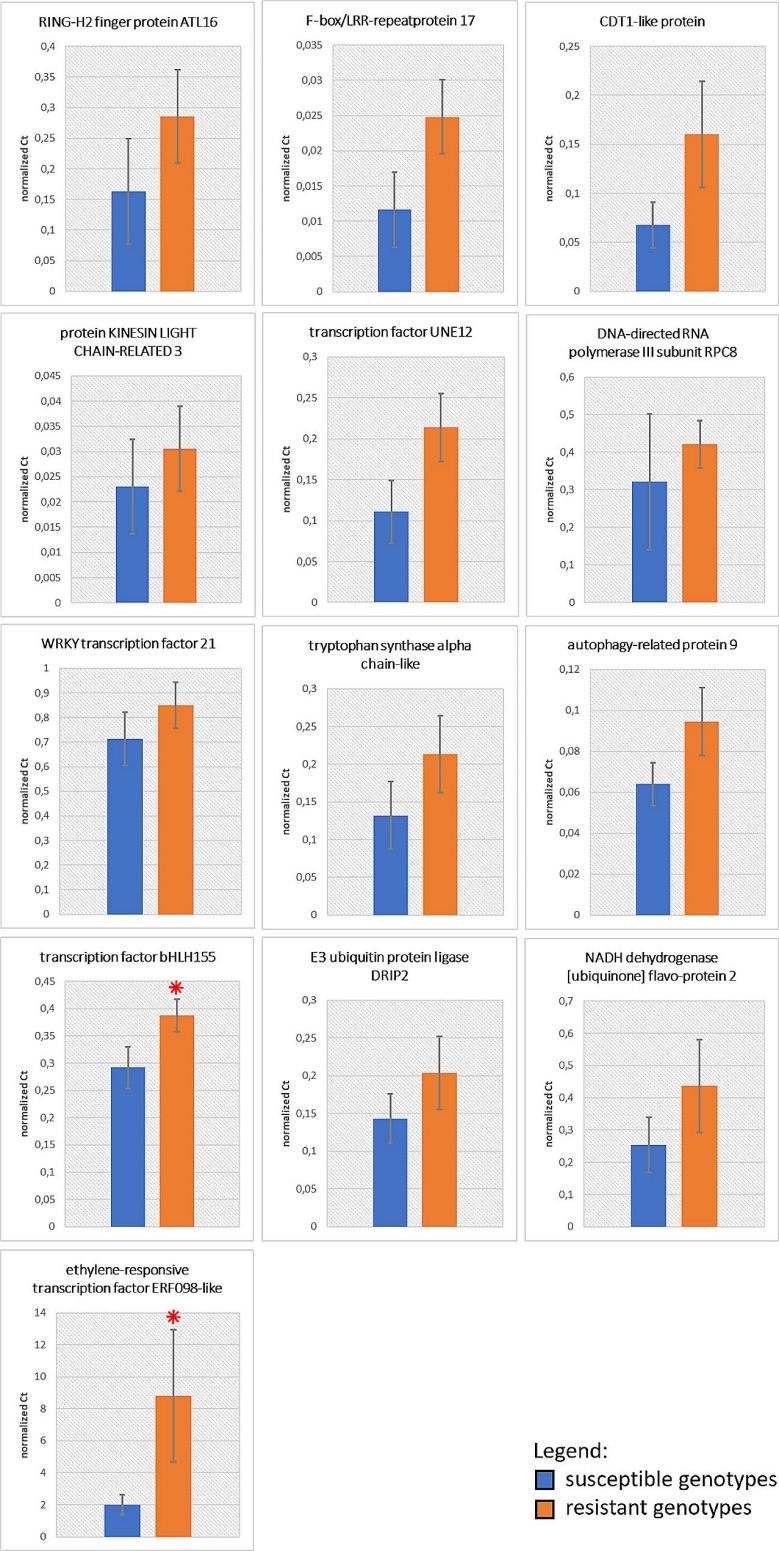
Results of gene expression analysis comparing susceptible (blue bars) and resistant genotypes (orange bars) for all genes related to response to biotic stress and/or synthesis of metabolites, according to the in silico annotation performed on linkage group 5, detected with QTL analysis

The involvement of the 13 candidate genes, detected through the *in silico* annotation of the QTL region, was further investigated by gene expression analysis through RT-qPCR. The analysis was performed on leaves of two groups of genotypes, chosen according to their percentage of damaged area subjected to spider mite infestation. In particular, genotypes ‘I179’ (1,3%), ‘I171’ (1,6%), ‘I92’ (1,9%), ‘I58’ (2,1%), ‘I163’ (2,2%), ‘I166’ (2,4%), were selected as resistant, while ‘I16’ (4,7%), ‘I102’ (8,3%), ‘I135’ (8,6%) and ‘I54’ (9,2%), were considered susceptible. For eleven of the thirteen genes tested, no significant differences were detected among the group of resistant and susceptible accessions. The only exceptions were represented by the transcription factor bHLH155-like and the ethylene-responsive transcription factor ERF098-like, whose expressions were respectively 1.3 (p value = 0.05) and 4.4 (p value = 0.04) times higher in the resistant genotypes than in the susceptible one (Fig. 7).

## Discussion

The *Tetranychidae* family is globally widespread and causes significant yield losses in a wide range of open-field and protected crops. Particularly, *T. urticae*, commonly known as the two-spotted spider mite, is one of the most destructive generalists, impacting citrus crops, with lemons and clementines being among the most affected. In protected environments, such as nurseries during propagation, the two-spotted spider mite infestations become even more severe, since mites take advantage of the protection against UV-A, to which they are highly susceptible [30]. The management of these pests is mainly based on the use of acaricides, with limitations due to the high cost of the products and mites’ ability to develop resistance. The Insecticide Resistance Action Commitee (IRAC) has already reported documented resistance to carbamates, avermectins, clofentezine, oganotin miticides, acequinocyl, and METI (Mitochondrial Complex Electron Transport Inhibitor) acaricides. Therefore, integrated control strategies, such as biological control with natural enemies, are recommended for mite management due to their low environmental impact and the ability to reduce chemical residues on fruits. Recent control strategies also emphasized host resistance showing that jasmonic acid (JA) signaling and the accumulation of flavonoids play a key-role in improving host resistance. Additionally, volatile organic compounds (VOCs) have been identified as essential factors in the attractiveness or repellence [31]. In this work, we developed and tested a novel detached-leaf phenotyping method on reference citrus genotypes. Our finding aligned with previous research, confirming the high resistance of sour orange to spider mite attacks [3]. Conversely trifoliate orange, citrange genotypes (Troyer and Carrizo), and ‘Cleopatra’ mandarin exhibited a higher degree of damage. Furthermore, we conducted a marker-trait association study to uncover the genetic basis of host resistance against *T. urticae*. To achieve this, we established a lemon segregating population crossing lemon ‘Interdonato’ and lemon ‘Femminello Siracusano 2Kr’. These two cultivars were selected for generating an inter-specific full-sib family, considering their widespread distribution in the Mediterranean area. Although both ‘Interdonato’ and ‘Femminello Siracusano 2Kr’ displayed an intermediate level of damage, the offsprings were characterized by a much wider phenotypic distribution. This is probably due to the rearrangement of susceptible recessive allele(s). Moreover, the focus on an inter-specific cross enables the use of seedlings characterized by superior performance as valuable pre-breeding material reducing the negative impact of linkage drag common in inter-specific crosses. In parallel with phenotyping, the lemon segregating population was also genotyped. Thanks to the availability of the lemon reference genome [25], we implemented the SPET approach for the first time in citrus. SPET is a high-throughput genotyping strategy known for its high reproducibility, capable of providing detailed genotype information on a customized panel of selected polymorphisms [17]. An association mapping analysis led to the identification of a QTL on chromosome 5 related to the percentage of damaged area. On the other side, no significant QTLs were detected for the number of survived mite, confirming that quantitative measurements are often more effective in capturing the overall phenotypic variability of a trait [32] As for the percentage of damaged area, the phenotypic distribution based on the most associated SNP suggested an additive genetic architecture of the trait (Fig. 5). The identified SNP can serve as a valuable tool for breeders in designing innovative breeding programs aimed at developing new lemon varieties that combine desirable agronomical traits with resistance to spider mite attacks. Furthermore, the gene ontology analysis performed on the genes within the QTL region on linkage group 5 highlighted the involvement of several genes associated with metabolites production and responses to biotic and abiotic stress. In a previous study in tomato, one QTL associated with resistance to spider mite was identified on chromosome 2. This was accomplished by using a recombinant inbred line population derived from a cross between the susceptible *Solanum lycopersicum* L. and the wild tomato *S. pimpinellifolium* [33]. Moreover, in maize, a QTL study was performed on three segregating populations sharing the same susceptible parent. In each of these populations, a significant signal was detected on chromosome 1 and 6, where several genes encoding benzoxazinoids (BX10, BX11 and BX12) were identified [34]. This finding reinforces the hypothesis that benzoxazinoids, which are glucoside conjugates acting as defensive compounds widespread in grasses, play a role in plant defenses since implicated in antibiosis to *T. urticae* [35].

The transcriptomic analysis on the genes underlying the QTL interval enabled the detection of two transcription factors (the ERF098-like and bHLH-155) showing a significant differential expression between the two groups of individuals selected. Several studies conducted on *Arabidopsis thaliana* indicate that ethylene-response factors (ERFs) are transcription factors that regulate the signaling cascade of a wide number of downstream genes related to stress responses and plant development, such as heat stress response [36], abscisic acid response [37], and tolerance to salt by the transcriptional activation of ascorbic acid synthesis [38]. The crucial role of ERF transcription factors is further highlighted by investigations into the ERF gene family in *A. thaliana* and in *Oryza sativa* L. subsp. *japonica* using a GWAS approach. This led to the identification of 122 and 139 ERF family genes in *A. thaliana* and in *Oryza sativa* L. subsp. *japonica* respectively [39]. As for the other candidate genes, several showed a differential, though not significant, differential expression between the two groups and have been previously recognized in other crops for their role in plant immunity against biotic and abiotic stresses. For instance, E3 ubiquitin ligase is crucial in the ubiquitination process and signaling during PAMP-Triggered Immunity (PTI) and Effector-Triggered Immunity [40]. Similarly, NADH dehydrogenase, when overexpressed in rice, leads an increased resistance towards *Xanthomonas oryzae* pv. *oryzae* due to the induction of reactive oxygen species (ROS) accumulation and the enhancement of pathogen-resistance genes [29].

## Conclusions

Given the observations in model plants and the results of the expression analysis in this study, we hypothesize that the ERF098-like gene identified in lemon could play a positively role in tolerance to spider mite attack by activating the plant defense response. Even though further analysis could provide a conclusive evidence of the involvement of the proposed candidate gene in enhancing tolerance towards spider mite attack, the identified polymorphism on linkage group 5 holds promise as tools for marker-assisted selection, speeding up breeding programs, and enabling the early selection of new varieties with desirable traits. Until now, the availability of markers to support citrus breeding has been limited, primarily focusing on traits such as Alternaria brown spot resistance [41, 42], *CTV* resistance [43], anthocyanin pigmentation [44], male sterility, and polyembryony [41, 45]. The introduction of new molecular markers associated with desirable traits, particularly those related to resistance to biotic stress and fruit quality, could significantly advance citrus genetic improvement. In this context, the development of new lemon varieties with enhanced resistance to spider mite is anticipated to revolutionize sustainable pest management practices in lemon orchards.

### Electronic supplementary material

Below is the link to the electronic supplementary material.

Supplementary Material 1

Supplementary Material 2

Supplementary Material 3

Supplementary Material 4

## Data Availability

Data is provided within the manuscript or supplementary information files.
