# Supplementary material for: QTL analysis on a lemon population provides novel insights on the genetic regulation of the tolerance to the two-spotted spider mite attack
Source: BMC Plant Biol. 2024 Jun 7;24:509. doi: 10.1186/s12870-024-05211-4 (PMC11157791; doi:10.1186/s12870-024-05211-4)
Supplement: Supplementary file 2 — Supplementary Material 2 [file 12870_2024_5211_MOESM2_ESM.docx]

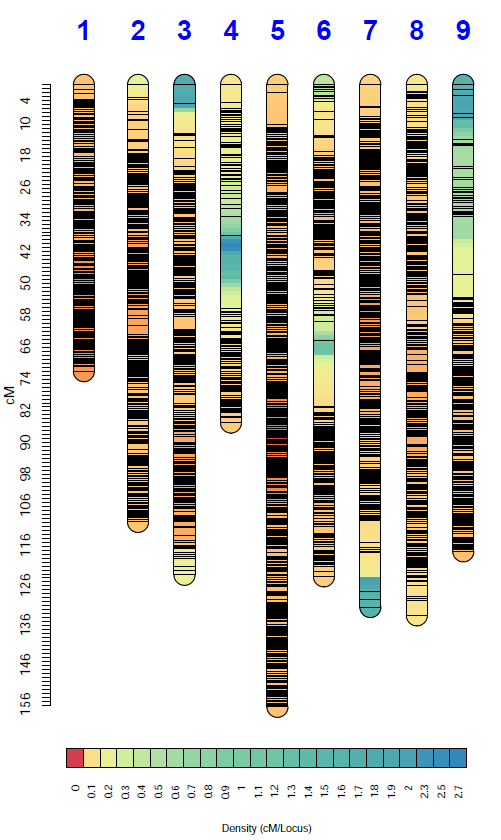


Additional file 3. Integrated genetic map generated employing one interspecific (‘Interdonato’ x ‘Femminello Siracusano 2kr’) and two interspecific segregating populations (*Citrus latipes* x ‘Femminello Siracusano 2kr’ and *Citrus clementina* x ‘Femminello Siracusano 2kr’).
