# Supplementary figures and images for: QTL analysis on a lemon population provides novel insights on the genetic regulation of the tolerance to the two-spotted spider mite attack
Source: BMC Plant Biol. 2024 Jun 7;24:509. doi: 10.1186/s12870-024-05211-4 (PMC11157791; doi:10.1186/s12870-024-05211-4)

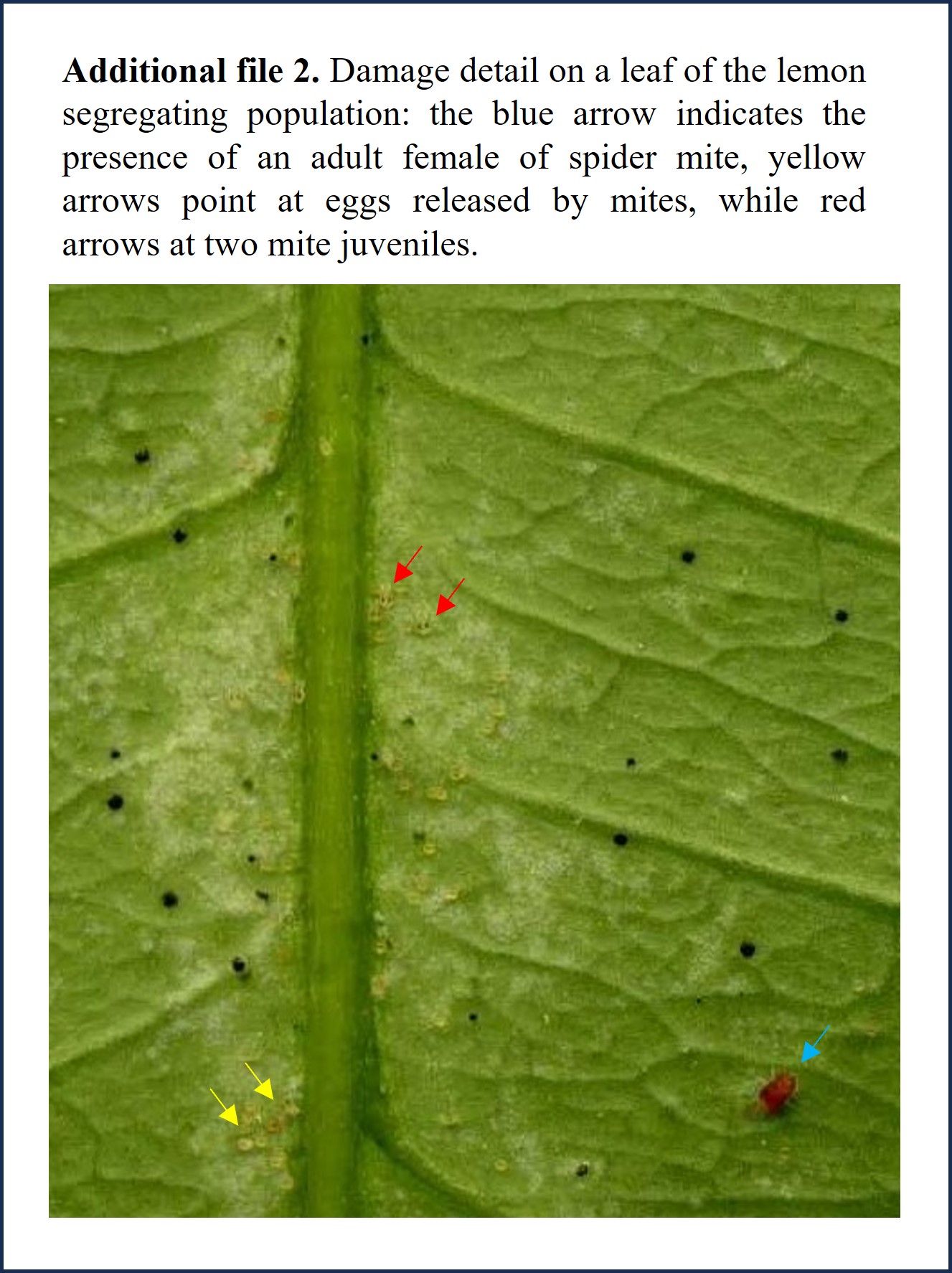

Supplement: Supplementary file 3 — Supplementary Material 3 [file 12870_2024_5211_MOESM3_ESM.jpg]
